# Objectively determined physical activity levels of primary school children in south-west Germany

**DOI:** 10.1186/1471-2458-13-895

**Published:** 2013-09-28

**Authors:** Sarah Kettner, Susanne Kobel, Nanette Fischbach, Clemens Drenowatz, Jens Dreyhaupt, Tamara Wirt, Benjamin Koch, Jürgen Michael Steinacker

**Affiliations:** 1 Sports- and Rehabilitation-Medicine, Research Group “Join the Healthy Boat – Primary School”, Ulm University, Frauensteige 6 - House 58 / 33 89075 Ulm, Germany; 2 Institute of Epidemiology and Medical Biometry, Ulm University, Ulm, Germany

**Keywords:** Inactivity, Energy expenditure, Youth, Body weight, Exercise

## Abstract

**Background:**

Only a small proportion of children and adolescents meet current recommendations of at least 60 min of moderate to vigorous physical activity (MVPA) daily. Most of the available data, however, relies on subjective reports; there is limited objective data on physical activity (PA) levels in German primary school children. The purpose of this study, therefore, was to accurately determine how much time children spend undertaking different intensities of PA and being sedentary during weekdays and weekend using objective assessment tools. Gender-specific and age-related differences were examined along with differences between normal weight and overweight/obese children.

**Methods:**

Children’s height and weight were measured according to standard procedures and objective PA measurements were determined in a sub-cohort of 384 primary school children (20% of the whole cohort), participating in a large school-based intervention study in south-west Germany (n = 1947). Baseline data collection occurred on six consecutive days, including weekend days, using multi-sensor accelerometry (Actiheart, CamNtech Ltd., Cambridge UK). 318 children (7.1 ± 0.6 years, male: 50%, first grade: 51%) provided data for at least 3 days including one weekend day. According to the amount of energy expended, defined as metabolic equivalents (METs), different activity intensities were categorised as follows: sedentary < 1.5 METs; light = 1.5-3.0 METs; moderate = 3.0-6.0 METs, and vigorous > 6.0 METs.

**Results:**

Average wear time was 1403 ± 94 min/day. Children spent 808 ± 97 min/day being sedentary; 497 ± 72 min/day in light; 128 ± 54 min/day in moderate, and 8 ± 10 min/day in vigorous intensity. 48% of children met the current MVPA guidelines. MVPA was significantly higher on weekdays compared to weekend days (144 ± 66 vs. 113 ± 66 min/day; p < 0.001). Furthermore, boys displayed higher MVPA levels compared to girls (164 ± 57 vs. 106 ± 50 min/day; p < 0.001).

**Conclusion:**

Measured objectively, less than half of primary school children in the study met current PA recommendations, emphasising the necessity for early intervention to promote PA. Consistent with previous research, PA levels were higher in boys and during weekdays. These results indicate that PA levels of girls should especially be promoted in primary schools and that parents should be more involved in interventions to improve PA, particularly during weekends.

## Background

It is well documented that sufficient physical activity (PA) is essential for childhood development and is highly relevant to health [1]. It is assumed that PA levels are established at a young age [2,3] and that an active lifestyle during childhood facilitates sufficient PA during adulthood [4-6]. Insufficient PA has been associated with an increased risk of obesity [1,7] and associated co-morbidities such as metabolic or cardiovascular diseases [8,9]. Besides biological reasons, declining PA levels have been predominantly attributed to social and cultural changes. In particular, current lifestyles promote sedentary routines due to a reduction in active transport, physical education at schools or organised sports [4,10].

The World Health Organisation recommends that children should engage in at least 60 min of moderate physical activity (moderate to vigorous physical activity (MVPA)) per day [11].

Based on self-report, it has been suggested that approximately 30-40% of children and adolescents between 2 and 18 years of age meet these recommendations [12]. In the German Health Interview and Examination Survey for Children and Adolescents (KiGGS), less than 20% of children aged 7–10 years reported sufficient MVPA. Furthermore, one in four children did not engage in regular sport and one in eight children did not participate in any sport [13,14]. In German adolescents, only 14% of girls and 20% of boys undertook at least 60 min of MVPA [15]. However, the accuracy of such subjective reports on PA in children especially remains questionable, due to their sporadic and unstructured behavioural patterns [16]. Consequently, pedometers or accelerometers have been introduced in order to accurately detect and measure children’s PA patterns [12]. Data from these studies would suggest that in children across different European countries only 4.6% of girls and 16.8% of boys aged 10–12 years meet current PA recommendations [17]. In Germany, data regarding objectively measured PA is limited, particularly in primary school children in first and second grade.

The purpose of the present study, therefore, was to objectively determine the amount of children’s PA and sedentary time with a multi-sensor accelerometer. In addition, differences in PA patterns between weekdays and weekends together with differences in PA between boys and girls were examined. Furthermore, age-related differences in PA and variances between normal weight and overweight/obese children were examined.

## Methods

### Study population

Baseline data of a sub-sample of the *Baden-Württemberg* study, which evaluated a large school-based health promotion programme *“Join the Healthy Boat – Primary School”* in south-west Germany, was used. Study design and protocol of the *Baden-Württemberg* study has been described in detail elsewhere [18]. Parental consent and child assent were obtained prior to data collection. The study was approved by the institutional ethics committee of Ulm University and is registered at the German Clinical Trials 127 Register (DRKS00000494).

Data collection occurred between September and November 2010, in a sample of 1947 children. For logistical reasons (distances between schools and scope of measurements of the *Baden-Württemberg* study) objective PA assessments were only carried out in the region of Ulm. A total number of 703 children in 32 schools and 56 classes (first and second grade) were available in this area. 62% of parents (n = 433) provided written informed consent for their children to wear the multi-sensor accelerometer for six consecutive days.

**Figure 1:**
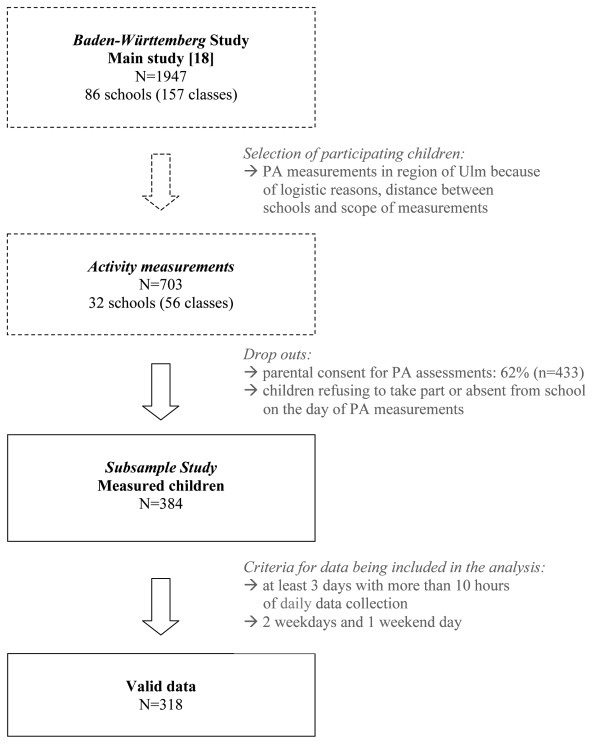
**Flow-chart of activity measurement procedure for *Baden-Württemberg *study.**

In order to be eligible to participate, a minimum of 5 children from each school were required to provide consent. The maximum number of parental consents obtained was 22 children. Due to children being absent from school on the day of the visit or refusing to take part in measurement of PA, a sub-sample of 384 children (20% of the whole cohort; n = 1947) was assessed. 83% of children (n = 318; 7.1 ± 0.6 years; boys: 50%; first grade: 51%) provided valid data of at least 3 days with more than 10 hours of daily wear time (Figure 1).

### Anthropometric measurements

Height and weight were measured according to standard procedures with children wearing only underwear and no shoes. Height was measured to the nearest 0.1 cm with a stadiometer (Seca 213, weighing and measurement systems, Hamburg, Germany) and weight was measured to the nearest 0.05 kg, using calibrated electronic scales (Seca 862, weighing and measurement systems, Hamburg, Germany). Body mass index (BMI) was calculated (kg/m^2^) and converted to BMI percentiles (BMIPCT) using German reference values [19]. Weight status has been subsequently classified as overweight/obese (≥ 90 percentile), normal weight (≥ 10 to < 90 percentile), and underweight (< 10 percentile). In addition, weight status was calculated according to international reference values [20] with corresponding results shown as Table 1.

**Table 1:** Descriptive characteristics of total sample, boys and girls

	**Total sample**	**Boys**	**Girls**
	**[n = 318]**	**[n = 159]**	**[n = 159]**
Age [yrs]	7.1 ± 0.6 [5.5–9.9]	7.1 ± 0.6 [6.0–9.9]	7.1 ± 0.6 [5.5–9.3]
Height [cm]	123.7 ± 6.1 [104.6–141.2]	124.0 ± 6.2 [106.4–141.2]	123.4 ± 6.1 [104.6–139.3]
Weight [kg]	24.6 ± 4.9 [15.60–47.00]	25.0 ± 5.0 [15.60–47.00]	24.2 ± 4.7 [16.65–45.50]
Weight status [%]			
International ref [20]			
- Overweight	9.5	10.1	8.9
- Obese	3.5	3.1	3.8

Values are mean ± SD and percentage of overweight/obese children [20].

### Physical activity measurements

A multi-sensor accelerometer (Actiheart, CamNtech Ltd., Cambridge UK) was fitted to each child’s chest using two electrodes and additionally protected with eudermic plasters. The Actiheart is a lightweight (8 g) multi-sensor accelerometer combining measurements of acceleration and heart rate (HR) to determine PA [21]. The participants were asked to wear the multi-sensor device for six consecutive days (à 24 h) including two weekend days. The recording interval was set to 15 sec. Parents were informed about the use and care of the tool in case the sensor was disconnected from the electrodes.

Energy expenditure, defined as metabolic equivalents (METs), was estimated via the captive Actiheart software (Version 4.0.73) [22], using a branched model approach previously validated in children [23]. Age, height, body weight and gender were used in addition to heart rate and accelerometry data to estimate the MET level. Data was classified as sedentary (< 1.5 METs); light (1.5-3.0 METs); moderate (3.0-6.0 METs), and vigorous (> 6.0 METs) [24].

To be included in the analysis, at least 3 days with more than 10 hours of daily data collection, including 2 weekdays and 1 weekend day, were required. Trost et al. (2000) indicate that 4 and 5 days of recording physical activity would be necessary to obtain a reliability of 0.80 in children [25]. Furthermore, to extrapolate the available days to a full week, a ratio of 5:2 for weekdays and weekend days was used. First and last recording days were excluded from analysis. In order to meet current PA guidelines, 60 min of MVPA was required for every single day of PA assessment.

In addition, PA was collected via parental questionnaire, whether the children engage in any sporting activities or participate in sports clubs. Parents were also asked on how many days per week their child achieves at least 60 min of MVPA.

### Socio-demographic data

Socio-demographic data was assessed via a parental questionnaire. Parental education was classified according to CASMIN [26], which was determined by the highest level of two parents or the level of a single parent. Due to the low number of cases at primary education level, primary and secondary education levels were combined into a single group (defined as low education level) while tertiary education level was considered to be high education level. Where there was at least one parent born abroad or speaking a foreign language during the first years of a child’s life, background was defined as “migration”.

### Statistical analysis

Descriptive statistics were calculated (mean values and standard deviations). Group differences between means were analysed with independent t-test and chi^2^-test was used to analyse group differences with categorical variables, respectively. Repeated measures ANOVA was used to examine differences between weekdays and weekend days. Furthermore, group differences between boys and girls were examined using ANCOVA adjusting for age and BMIPCT. Similarly, differences between first and second grade children were analysed, adjusting for gender and BMIPCT. To determine differences by weight status ANCOVA, adjusting for gender and age has been used. Underweight children (n = 26; 8%) were excluded from analysis. Statistical analyses were performed using PASW 19.0 (Predictive Analytics Software, SPSS Inc, Chicago, IL, US), with a level of significance set at p ≤ 0.05.

## Results

Between the sub-sample (n = 384) and the total study population there were no differences in descriptive characteristics (sex, age, height, body weight, BMI percentiles (BMIPCT), sports participation, migration background and parent education). Children from the 32 schools and 56 classes in the region of Ulm providing consent for the objective measurement did not differ from those refusing to participate in the objective measurement either. Concerning subjectively assessed PA behaviour via parental questionnaire no differences were found between the sub-sample and the total study population.

Descriptive characteristics are shown in Table 2. There were no differences in anthropometric characteristics between boys and girls. Almost 11% (n = 34) of the children were classified as overweight or obese, based on German reference values. The prevalence was slightly higher using international cut-off points [20] (Table 1). Average recording time was 1403 ± 94 min/day. There were no differences in wearing time between boys and girls, first and second grade children nor between normal weight and overweight children. Table 3 displays the time spent in different activity intensities and the percentage of children achieving MVPA guidelines. Less than half of the children (48%) met current PA recommendations. Including sleep, most of the time was spent being sedentary (56.1%) with roughly one third in light activities (34.5%). Only 9.4% of the time accounted for MVPA, mainly in moderate intensity (8.9%).

**Table 2:** Descriptive characteristics of total sample, boys and girls

	**Total sample**	**Boys**	**Girls**
	**[n = 318]**	**[n = 159]**	**[n = 159]**
Age [yrs]	7.1 ± 0.6 [5.5–9.9]	7.1 ± 0.6 [6.0–9.9]	7.1 ± 0.6 [5.5–9.3]
Height [cm]	123.7 ± 6.1 [104.6–141.2]	124.0 ± 6.2 [106.4–141.2]	123.4 ± 6.1 [104.6–139.3]
Weight [kg]	24.6 ± 4.9 [15.60–47.00]	25.0 ± 5.0 [15.60–47.00]	24.2 ± 4.7 [16.65–45.50]
BMIPCT	48.1 ± 28.0 [2–100]	50.4 ± 27.2 [2–100]	45.7 ± 28.6 [2–100]
Weight status [%]			
- Overweight	6.3	6.3	6.3
- Obese	4.4	5.0	3.8

Values are mean ± SD and percentage of overweight/obese children [20].

**Table 3:** Time spent in different intensities and percentage of children meeting PA guidelines

	**Total sample**	**Boys**	**Girls**	**p-value**
	**[n = 318]**	**[n = 159]**	**[n = 159]**	
**Total week [min/day]**				
Sedentary PA	808 ± 97	775 ± 86	841 ± 97	**p < 0.001**
Light PA	497 ± 72	501 ± 71	492 ± 73	p = 0.179
Moderate PA	128 ± 54	152 ± 51	103 ± 46	**p < 0.001**
Vigorous PA	8 ± 10	12 ± 11	3 ± 6	**p < 0.001**
MVPA	135 ± 61	164 ± 57	106 ± 50	**p < 0.001**
**Weekend [min/day]**				
Sedentary PA	851 ± 110	820 ± 102	883 ± 108	**p < 0.001**
Light PA	476 ± 80	481 ± 78	470 ± 82	p = 0.154
Moderate PA	108 ± 62	131 ± 60	85 ± 54	**p < 0.001**
Vigorous PA	5 ± 8	8 ± 9	2 ± 4	**p < 0.001**
MVPA	113 ± 66	139 ± 65	87 ± 56	**p < 0.001**
**Weekdays [min/day]**				
Sedentary PA	791 ± 104	757 ± 92	825 ± 105	**p < 0.001**
Light PA	505 ± 79	509 ± 78	501 ± 80	p = 0.258
Moderate PA	135 ± 58	160 ± 56	111 ± 50	**p < 0.001**
Vigorous PA	9 ± 12	14 ± 13	3 ± 8	**p < 0.001**
MVPA	144 ± 66	174 ± 64	114 ± 55	**p < 0.001**
**≥ 60 min MVPA per day**				
Meeting recommendations	48%	68%	28%	

Values are mean ± SD. P-value based on ANCOVA, adjusted for age and BMIPCT.

Values are mean ± SD and percentage of overweight/obese children [20].

**Figure 2:**
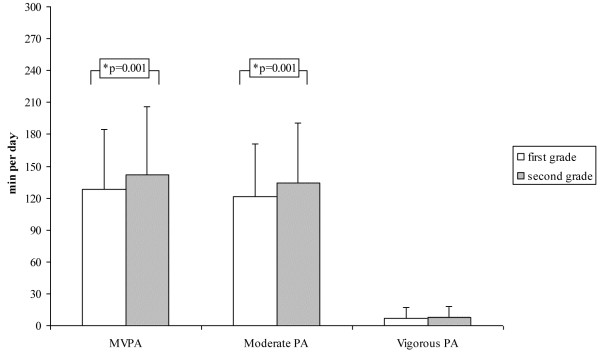
**Different PA levels of first grade and second grade children; values are mean ± SD.** P-value based on ANCOVA, adjusted for gender and BMIPCT.

Girls spent more time in sedentary activities (841 ± 97 min/day vs. 775 ± 86 min/day for girls and boys, respectively; p < 0.001), while boys were more active than girls in MVPA (164 ± 57 min/day vs. 106 ± 50 min/day, respectively; p < 0.001). Consequently, more boys met PA recommendations compared to girls (68% vs. 28%). Moreover, there was a difference between activity behaviour during the week and weekend. Higher PA levels were observed during the week [F_MVPA_(1,317) = 91.6; p < 0.001] whilst more time was spent being sedentary during the weekend [F_SED_(1,317) = 134.3; p < 0.001]. Children who recently started school (first grade) spent more time in light intensity than children in second grade (506 ± 73 vs. 488 ± 70 min per day, respectively; p = 0.024), whereas second grade children spent more time undertaking moderate activities, resulting in more time spent in MVPA (Figure 2). No difference was found for sedentary activities between first and second grade children.

**Figure 3:**
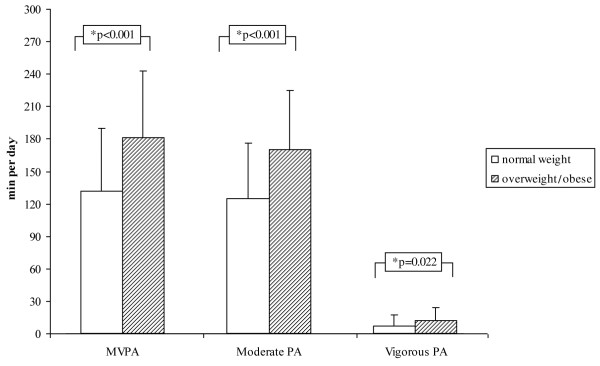
**Different PA levels of normal weight and overweight/obese children; values are mean ± SD.** P-value based on ANCOVA, adjusted for gender and age; *underweight children (n = 26) were excluded from analyses.

In addition, there was no difference in sedentary time between normal weight and overweight/obese children but normal weight children spent more time doing light activities (498 ± 68 vs. 467 ± 66 min per day, respectively; p = 0.023) than their overweight/obese counterparts. However, children categorised as overweight or obese spent more time in moderate and vigorous intensities than normal weight children, resulting in higher MVPA levels (Figure 3). Similar results occurred when using IOTF (International Obesity Task Force) cut-off points for overweight and obesity (Table 4). Interactions between gender and weight status as well as interactions between gender and first and second grade children were not significant.

**Table 4:** **Differences between normal weight and overweight/obese children**[20]**in time spent in different PA intensities and percentage of children meeting PA guidelines**

	**Total sample**	**Normal weight**	**Overweight/obese**	p-value*
	**[n = 318]**	**[n = 241]**	**[n = 41]**	
**Total Week [min/day]**				
Sedentary PA	808 ± 97	810 ± 95	793 ± 92	p = 0.264
Light PA	497 ± 72	497 ± 68	471 ± 67	**p = 0.049**
Moderate PA	128 ± 54	125 ± 52	164 ± 55	**p < 0.001**
Vigorous PA	8 ± 10	8 ± 10	11 ± 12	**p = 0.019**
MVPA	135 ± 61	133 ± 58	176 ± 63	**p < 0.001**
**Weekend [min/day]**				
Sedentary PA	851 ± 110	856 ± 109	826 ± 108	p = 0.066
Light PA	476 ± 80	474 ± 79	463 ± 74	p = 0.625
Moderate PA	108 ± 62	105 ± 61	144 ± 60	**p < 0.001**
Vigorous PA	5 ± 8	5 ± 8	7 ± 10	**p = 0.023**
MVPA	113 ± 66	110 ± 65	151 ± 66	**p < 0.001**
**Weekdays [min/day]**				
Sedentary PA	791 ± 104	791 ± 102	779 ± 98	p = 0.520
Light PA	505 ± 79	507 ± 75	475 ± 74	**p = 0.021**
Moderate PA	135 ± 58	134 ± 55	173 ± 59	**p < 0.001**
Vigorous PA	9 ± 12	9 ± 12	13 ± 14	**p = 0.046**
MVPA	144 ± 66	142 ± 64	186 ± 69	**p < 0.001**
**≥ 60 min MVPA per day**				
Meeting recommendations	48%	46%	78%	

Values are mean ± SD. P-value based on ANCOVA, adjusted for gender and age; *underweight children (n = 36) were excluded from analyses.

## Discussion

The results of this study indicate that school children from south-west Germany spend over half of their day being sedentary (56.1%) and one third of their day undertaking light activities (34.5%). Less than half of these children are meeting the current PA recommendations of at least 60 min MVPA per day.

This reported percentage of children meeting current PA recommendations is comparable to Spanish children [27] while it is slightly higher than previously reported PA levels of 7-10-year-old German children, based on self-reported PA [13]. Riddoch et al. (2004), conversely, reported that 80% of 9-year-old children from a large Norwegian sample met current PA guidelines [28]. Overall, these results emphasise that the proportion of children meeting current PA recommendations varies enormously and differs between countries. Beets et al. (2011) reported a span of between 7% and 96% in pre-school children complying with PA recommendations [29]. These differences may be at least partially be explained by variations in the interpretation of guidelines, alternative methodologies and the utilisation of different cut-off points to determine MVPA when using accelerometry [12].

In the present study, children were less active during the weekend than during the week, despite weekend days offering children more available time for physical activities, such as playing outdoors. Similar results have been reported in German pre-school children aged 3–6 years and in 9 and 15 year old European and Canadian children aged 10–15 years [30-32].

The above findings are particularly relevant for the design of effective intervention programmes, as they indicate a need for a stronger involvement of parents in health promotion programmes. Sallis et al. (1991) argue that parents have an essential impact on their child’s PA [16] and several studies have indicated that children of more active parents are also likely to be more physically active [33,34]. In addition, parental encouragement for PA has been associated with positive effects on children’s PA [35]. For the promotion of PA, it is important to identify opportunities to families for being active, especially at the weekend. Furthermore, Cleland et al. (2008) emphasised the importance of time spent outdoors during the weekend as this was associated with higher MVPA in 10–12 year old children [36]. Children spending more time outdoors also displayed a lower prevalence of overweight. However, more research is needed to gain further clarity regarding the influence of parental factors on health-related parameters such as MVPA or sedentary behaviour in primary school children.

In the present study more than two thirds of boys achieved at least 60 min of MVPA per day, whilst only 28% of girls met current recommendations. Similar results showing higher PA in boys compared to girls have previously been reported worldwide, e.g. in the United States amongst 6–19 year olds from the representative National Health and Nutrition Examination Survey (NHANES), and in the European Youth Heart Study (EYHS) examining a representative sample of different countries in Europe of 9 and 15-year-olds [28,37].

Despite the fact that PA levels have been shown to decline with age [38,39], second grade children displayed higher activity levels then first graders in the current study. One reason may be that first graders have not yet adjusted to their new role as pupils or the increased sedentary time in the morning. In addition, sports club participation increases throughout childhood [14]. This was also shown in the present study where second graders spent more time in organised sports than first graders (data not shown), although these results were not statistically significant. In general, 75% of the children in the sub-sample in addition to the children in the main study participate in sports clubs. These results are consistent with the representative KiGGS study, where nearly three-quarters of the children participate in sports clubs [14].

Furthermore, the study revealed that overweight/obese children spent more time in MVPA compared to normal weight children. This is in contrast to previous studies examining PA patterns between obese and normal weight children and adolescents aged 5 to 17 years [40-42]. One explanation of this finding might be the fact that the current study relied on both heart rate and accelerometry to determine PA rather than relying individually on heart rate or accelerometry. Overweight children might have displayed higher heart rates at light activities, which may in error have been classified as moderate. In this study however, using only heart rate data no differences in PA intensities between normal weight and overweight/obese children were found. There was no difference in average movement counts per day between normal weight and overweight/obese children either (data not shown). Other studies have also reported higher PA in overweight or obese children [43,44] whilst some [45,46] did not show any association between body weight and PA. In addition to that differences in measurement tools and discrepancies in sample size of overweight/obese children may have contributed to these ambiguous findings.

Limitations in the present study need to be considered when interpreting the findings. Even though PA was assessed objectively, the utilisation of METs to differentiate between PA intensity levels might have led to a misrepresentation of PA in the study population. Additionally, an interval of 15 sec might be too long to accurately display children’s activity patterns as, typically, they engage in very short, highly variable, unstructured movements [4,47]. Bailey et al. (1995) showed that periods of low and moderate intensities in children took an average of only 6 sec and periods of vigorous intensity 3 sec [47]. These short bouts may not have been adequately captured with a long interval in the present study. Even though children were selected state-wide from study schools involving different rural and urban settings and different social structures, there could have been a selection bias due to the fact that only pupils of teachers, who were interested in participating in a school-based intervention programme, were considered. Nevertheless, a national sample (KiGGS study) showed only a marginally higher prevalence of 15% overweight and obese children aged 7–10 years [48]. A comparable overweight prevalence of about 13% was found in German children in the state of *Baden-Württemberg* at school entry [49]. The KiGGS study also showed a similar proportion of children with migration status, while the percentage of families with lower parental education was slightly higher compared to the present study [50,51].

At this time, this study only extrapolated data of objectively assessed PA over a period of 24 hours to detect PA behaviour of primary school children has been used. Further research is necessary to get a close understanding of PA behaviour over the whole day with excluding sleeping time to get more information of different aspects during the day. Finally, it should be considered that data collection took place in autumn and weather may have influenced PA levels. However, it was not possible to determine the influence of weather on PA patterns of children. For further investigations this factor should be included in the analysis.

## Conclusions

In summary, approximately just under half of primary school children in first and second grades meet current PA recommendations. Since sufficient PA in childhood contributes to an active lifestyle and other health-related parameters in later life, effective intervention programmes for the promotion of PA are required. The results of this study emphasise, in particular, low activity levels at the weekend. In order to achieve sufficient PA levels throughout the entire week, stronger parental and school involvement will be essential.

## Abbreviations

BMI: Body mass index; BMIPCT: Body mass index percentile; cm: Centimeter; e.g.: For example; HR: Heart rate; IOTF: International Obesity Task Force; kg: Kilogram; m2: Square meter; MET: Metabolic equivalent task; min: Minutes; MVPA: Moderate to vigorous physical activity; PA: Physical activity; PASW: Predictive analytics software; ref: Reference; SD: Standard deviation; sec: Seconds; vs.: Versus; yrs: Years.

## Competing interests

The authors declare that they have no competing interests.

## Authors’ contributions

SK (corresponding author): conceptional work, data collection, data analyses, manuscript draft, manuscript editing, submission. SK: conceptional work, data collection, data analyses, manuscript revision. NF: data analyses, manuscript revision. CD: data analyses, manuscript revision. JD: conceptional work, data analyses, manuscript revision. TW: data collection, data analyses, manuscript revision. BK: conceptional work, manuscript revision. JMS: manuscript revision. All authors read and approved the final manuscript.

## Pre-publication history

The pre-publication history for this paper can be accessed here:

http://www.biomedcentral.com/1471-2458/13/895/prepub
